# Supplementary material for: Machine Learning Accelerates Crystallization for Structure Determination
Source: Angew Chem Int Ed Engl. 2026 May 4;65(25):e1218503. doi: 10.1002/anie.1218503 (PMC13266958; doi:10.1002/anie.1218503)

checkCIF/PLATON report

Structure factors have been supplied for datablock(s) lcz043

THIS REPORT IS FOR GUIDANCE ONLY. IF USED AS PART OF A REVIEW PROCEDURE FOR PUBLICATION, IT SHOULD NOT REPLACE THE EXPERTISE OF AN EXPERIENCED CRYSTALLOGRAPHIC REFEREE.

No syntax errors found. CIF dictionary Interpreting this report

Datablock: lcz043

Bond precision:	C-C = 0.0081 A		Wavelength=1.54184
Cell:	a=16.2052(1)	b=17.7791(2)	c=24.0541(2)
	alpha=104.377(1)	beta=99.060(1)	gamma=111.762(1)
Temperature:	100 K		

	Calculated	Reported
Volume	5990.22(12)	5990.22(10)
Space group	P -1	P -1
Hall group	-P 1	-P 1
Moiety formula	2(C33 H21 Ag3 F18 N6 O5), C15 H3 Ag3 F18 N6, C18 H18 O5	3(C15 H3 Ag3 F18 N6), 3(C18 H18 O5)
Sum formula	C99 H63 Ag9 F54 N18 O15	C99 H63 Ag9 F54 N18 O15
Mr	3741.51	3741.50
Dx,g cm-3	2.074	2.074
Z	2	2
Mu (mm-1)	12.931	12.931
F000	3624.0	3624.0
F000’	3640.94	
h,k,lmax	20,22,29	20,21,29
Nref	23993	22504
Tmin,Tmax	0.265,0.274	0.592,1.000
Tmin’	0.170	

```
Correction method= # Reported T Limits: Tmin=0.592 Tmax=1.000
AbsCorr = MULTI-SCAN

Data completeness= 0.938            Theta(max)= 73.161

R(reflections)= 0.0431( 20440)               wR2(reflections)= 0.1160( 22504)

S = 1.034                 Npar= 1768
```

The following ALERTS were generated. Each ALERT has the format
test-name_ALERT_alert-type_alert-level.
Click on the hyperlinks for more details of the test.

Alert level C

```
PLAT029_ALERT_3_C _diffrn_measured_fraction_theta_full value Low .      0.970 Why?
PLAT042_ALERT_1_C Calc. and Reported MoietyFormula Strings  Differ     Please Check
              Calc: 2(C33 H21 Ag3 F18 N6 O5), C15 H3 Ag3 F18 N6, C
                    18 H18 O5
              Rep.: 3(C15 H3 Ag3 F18 N6), 3(C18 H18 O5)
PLAT213_ALERT_2_C Atom F01X            has ADP max/min Ratio .....         3.3 prolat
PLAT213_ALERT_2_C Atom F01Z            has ADP max/min Ratio .....         3.2 prolat
PLAT213_ALERT_2_C Atom F02C            has ADP max/min Ratio .....         3.5 prolat
PLAT213_ALERT_2_C Atom F02N            has ADP max/min Ratio .....         3.8 prolat
PLAT214_ALERT_2_C Atom F03M      (Anion/Solvent) ADP max/min Ratio         4.3 prolat
PLAT214_ALERT_2_C Atom F057      (Anion/Solvent) ADP max/min Ratio         4.2 prolat
PLAT220_ALERT_2_C NonSolvent   Resd 2  C   Ueq(max)/Ueq(min) Range         3.2 Ratio
PLAT221_ALERT_2_C Solv./Anion  Resd 3  F   Ueq(max)/Ueq(min) Range         5.2 Ratio
PLAT234_ALERT_4_C Large Hirshfeld Difference F04O     --C053     .        0.17 Ang.
PLAT241_ALERT_2_C High   'MainMol' Ueq as Compared to Neighbors of        O01N Check
PLAT250_ALERT_2_C Large U3/U1 Ratio for <U(i,j)> Tensor(Resd    3)         2.3 Note
PLAT342_ALERT_3_C Low Bond Precision on  C-C Bonds ..............     0.00808 Ang.
PLAT790_ALERT_4_C Centre of Gravity not Within Unit-Cell: Resd.  #           1 Note
              C33 H21 Ag3 F18 N6 O5
PLAT910_ALERT_3_C Missing FCF Reflection(s) Below Theta(Min)[Deg]=        3.67 Note
                1  0  0,  -1  1  0,   0  1  0,   0 -1  1,  -1  0  1,   0  0  1,
PLAT911_ALERT_3_C Missing FCF Refl Between Thmin & STh/L=    0.600         655 Report
              -15  5  1,   1 13  3, -12-10  6,  -9-10  6, -13 -9  6,  17 -3  6,
              -14 10  6, -10-12  7, -11-11  7, -12-10  7, -13 -9  7, -10 -9  7,
               17 -4  7, -13 18  7,  -9-13  8, -10-12  8, -11-11  8, -12-10  8,
              -13 -9  8, -10 -9  8, -13 -8  8, -14 -7  8, -15 -6  8,  16 -2  8,
               15  0  8,  -7-15  9,  -8-14  9,  -9-13  9, -10-12  9, -11-11  9,
                   (  625 More Missing: see the .ckf listing file)
PLAT972_ALERT_2_C Check Calcd Resid. Dens.  0.80Ang From Ag06          -1.70 eA-3
PLAT972_ALERT_2_C Check Calcd Resid. Dens.  0.84Ang From Ag03          -1.69 eA-3
PLAT972_ALERT_2_C Check Calcd Resid. Dens.  0.85Ang From Ag01          -1.68 eA-3
PLAT972_ALERT_2_C Check Calcd Resid. Dens.  0.83Ang From Ag05          -1.66 eA-3
PLAT972_ALERT_2_C Check Calcd Resid. Dens.  0.88Ang From Ag07          -1.65 eA-3
PLAT972_ALERT_2_C Check Calcd Resid. Dens.  0.89Ang From Ag08          -1.61 eA-3
PLAT972_ALERT_2_C Check Calcd Resid. Dens.  0.91Ang From Ag07          -1.60 eA-3
PLAT972_ALERT_2_C Check Calcd Resid. Dens.  0.78Ang From Ag03          -1.59 eA-3
PLAT972_ALERT_2_C Check Calcd Resid. Dens.  0.82Ang From Ag04          -1.58 eA-3
PLAT972_ALERT_2_C Check Calcd Resid. Dens.  0.83Ang From Ag09          -1.56 eA-3
PLAT972_ALERT_2_C Check Calcd Resid. Dens.  0.93Ang From Ag08          -1.55 eA-3
```

```
PLAT972_ALERT_2_C Check Calcd Resid. Dens.  0.81Ang From Ag06          -1.54 eA-3
PLAT972_ALERT_2_C Check Calcd Resid. Dens.  0.82Ang From Ag02          -1.52 eA-3
PLAT972_ALERT_2_C Check Calcd Resid. Dens.  0.84Ang From Ag07          -1.51 eA-3
```

Alert level G

```
PLAT007_ALERT_5_G Number of Unrefined Donor-H Atoms .............          3 Report
              H00Z  H01L  H01M
PLAT083_ALERT_2_G SHELXL Second Parameter in WGHT  Unusually Large      19.87 Why ?
PLAT152_ALERT_1_G The Supplied and Calc. Volume s.u. Differ by ...          2 Units
PLAT154_ALERT_1_G The s.u.'s on the Cell Angles are Equal ..(Note)      0.001 Degree
PLAT242_ALERT_2_G Low    'MainMol' Ueq as Compared to Neighbors of       C03J Check
PLAT242_ALERT_2_G Low    'MainMol' Ueq as Compared to Neighbors of       C03V Check
PLAT242_ALERT_2_G Low    'MainMol' Ueq as Compared to Neighbors of       C03X Check
PLAT242_ALERT_2_G Low    'MainMol' Ueq as Compared to Neighbors of       C04Y Check
PLAT242_ALERT_2_G Low    'MainMol' Ueq as Compared to Neighbors of       C051 Check
PLAT242_ALERT_2_G Low    'MainMol' Ueq as Compared to Neighbors of       C059 Check
PLAT242_ALERT_2_G Low    'MainMol' Ueq as Compared to Neighbors of       C03I Check
PLAT242_ALERT_2_G Low    'MainMol' Ueq as Compared to Neighbors of       C040 Check
PLAT242_ALERT_2_G Low    'MainMol' Ueq as Compared to Neighbors of       C044 Check
PLAT242_ALERT_2_G Low    'MainMol' Ueq as Compared to Neighbors of       C046 Check
PLAT242_ALERT_2_G Low    'MainMol' Ueq as Compared to Neighbors of       C04C Check
PLAT242_ALERT_2_G Low    'MainMol' Ueq as Compared to Neighbors of       C04L Check
PLAT242_ALERT_2_G Low    'MainMol' Ueq as Compared to Neighbors of       C02M Check
PLAT242_ALERT_2_G Low    'MainMol' Ueq as Compared to Neighbors of       C035 Check
PLAT242_ALERT_2_G Low    'MainMol' Ueq as Compared to Neighbors of       C04D Check
PLAT242_ALERT_2_G Low    'MainMol' Ueq as Compared to Neighbors of       C04Z Check
PLAT242_ALERT_2_G Low    'MainMol' Ueq as Compared to Neighbors of       C053 Check
PLAT242_ALERT_2_G Low    'MainMol' Ueq as Compared to Neighbors of       C05B Check
PLAT434_ALERT_2_G Short Inter HL..HL Contact F00E     ..F01Z     .       2.74 Ang.
                                                -1+x,y,z  =     1_455 Check
PLAT434_ALERT_2_G Short Inter HL..HL Contact F00T     ..F029     .       2.82 Ang.
                                                 1+x,1+y,z  =     1_665 Check
PLAT434_ALERT_2_G Short Inter HL..HL Contact F00Y     ..F01C     .       2.75 Ang.
                                                 1-x,-y,1-z  =     2_656 Check
PLAT434_ALERT_2_G Short Inter HL..HL Contact F01V     ..F02C     .       2.67 Ang.
                                                 2-x,1-y,-z  =     2_765 Check
PLAT434_ALERT_2_G Short Inter HL..HL Contact F02B     ..F02B     .       2.83 Ang.
                                                 1-x,-y,1-z  =     2_656 Check
PLAT720_ALERT_4_G Number of Unusual/Non-Standard Labels ..........        258 Note
              Ag01    Ag02    Ag03    Ag04    Ag05    Ag06    Ag07    Ag08
              Ag09    F00A    F00B    F00C    F00D    F00E    F00F    F00G
              F00H    F00I    F00J    F00K    O00L    O00M    F00N    F00O
              F00P    F00Q    F00R    F00S    F00T    F00U    F00V    F00W
              F00X    F00Y    O00Z    H00Z    F010    O011    F012    O013
              F014    O015    F016    N017    F018    N019    N01A    F01B
              F01C    N01D    F01E    N01F    N01G    N01H    N01I    N01J
              N01K    O01L    H01L    O01M    H01M    O01N    N01O    N01P
              N01Q    O01R    F01S    N01T    F01U    F01V    F01W    F01X
              O01Y    F01Z    N020    N021    F022    F023    N024    N025
              O026    F027    F028    F029    O02A    F02B    F02C    F02D
              C02E    C02F    C02G    C02H    O02I    O02J    C02K    C02L
              C02M    F02N    C02O    C02P    C02Q    H02Q    C02R    H02R
              C02S    H02S    C02T    H02T    F02U    C02V    H02V    C02W
              C02X    C02Y    H02Y    C02Z    F030    C031    C032    C033
              F034    C035    C036    F037    C038    H038    C039    H039
              C03A    C03B    C03C    C03D    H03D    C03E    H03E    C03F
```

```
               H03F    C03G    H03G    C03H    H03H    C03I    C03J    C03K
               C03L    F03M    C03N    H03N    C03O    C03P    H03P    C03Q
               C03R    C03S    C03T    C03U    C03V    C03W    H03A    H03B
               H03C    C03X    C03Y    H03Y    C03Z    C040    C041    C042
               H042    C043    C044    C045    H045    C046    C047    H04A
               H04B    H04C    C048    C049    C04A    C04B    H04H    C04C
               C04D    C04E    H04M    C04F    H04F    C04G    C04H    H04N
               C04I    H04I    H04K    H04L    C04J    H04J    C04K    H04O
               C04L    C04M    H04P    C04N    F04O    C04P    H04D    H04E
               H04G    C04Q    H04Q    C04R    H04R    F04S    C04T    C04U
               C04V    H04V    C04W    H04W    C04X    H04X    C04Y    C04Z
               C050    C051    C052    H052    C053    C054    C055    H055
               C056    F057    C058    H05A    H05B    H05C    C059    C05A
               H05D    C05B    C05C    H05H    H05I    H05J    C05D    H05K
               H05L    H05M    C05E    H05N    H05O    H05P    C05F    H05E
               H05F    H05G
PLAT790_ALERT_4_G Centre of Gravity not Within Unit-Cell: Resd.  #          2 Note
               C33 H21 Ag3 F18 N6 O5
PLAT883_ALERT_1_G Absent Datum for _atom_sites_solution_primary ..     Please Do !
PLAT912_ALERT_4_G Missing # of FCF Reflections Above STh/L=  0.600        818 Note
PLAT941_ALERT_3_G Average HKL Measurement Multiplicity ..........        4.6 Low
PLAT969_ALERT_5_G The 'Henn et al.' R-Factor-gap value ..........      5.237 Note
               Predicted wR2: Based on SigI**2  2.22 or SHELX Weight 11.22
PLAT978_ALERT_2_G Number C-C Bonds with Positive Residual Density.          0 Info
```

```
   0 ALERT level A = Most likely a serious problem - resolve or explain
   0 ALERT level B = A potentially serious problem, consider carefully
  31 ALERT level C = Check. Ensure it is not caused by an omission or oversight
  34 ALERT level G = General information/check it is not something unexpected

   4 ALERT type 1 CIF construction/syntax error, inconsistent or missing data
  49 ALERT type 2 Indicator that the structure model may be wrong or deficient
   5 ALERT type 3 Indicator that the structure quality may be low
   5 ALERT type 4 Improvement, methodology, query or suggestion
   2 ALERT type 5 Informative message, check
```

Validation response form

Please find below a validation response form (VRF) that can be filled in and pasted into your CIF.

```
# start Validation Reply Form
_vrf_PLAT029_lcz043
;
PROBLEM: _diffrn_measured_fraction_theta_full value Low .      0.970 Why?
RESPONSE: ...
;
_vrf_PLAT042_lcz043
;
PROBLEM: Calc. and Reported MoietyFormula Strings  Differ     Please Check
RESPONSE: ...
;
_vrf_PLAT213_lcz043
;
PROBLEM: Atom F01X            has ADP max/min Ratio .....        3.3 prolat
RESPONSE: ...
```

```
;
_vrf_PLAT214_lcz043
;
PROBLEM: Atom F03M      (Anion/Solvent) ADP max/min Ratio        4.3 prolat
RESPONSE: ...
;
_vrf_PLAT220_lcz043
;
PROBLEM: NonSolvent   Resd 2  C   Ueq(max)/Ueq(min) Range        3.2 Ratio
RESPONSE: ...
;
_vrf_PLAT221_lcz043
;
PROBLEM: Solv./Anion  Resd 3  F   Ueq(max)/Ueq(min) Range        5.2 Ratio
RESPONSE: ...
;
_vrf_PLAT234_lcz043
;
PROBLEM: Large Hirshfeld Difference F04O     --C053     .       0.17 Ang.
RESPONSE: ...
;
_vrf_PLAT241_lcz043
;
PROBLEM: High   'MainMol' Ueq as Compared to Neighbors of       O01N Check
RESPONSE: ...
;
_vrf_PLAT250_lcz043
;
PROBLEM: Large U3/U1 Ratio for <U(i,j)> Tensor(Resd    3)        2.3 Note
RESPONSE: ...
;
_vrf_PLAT342_lcz043
;
PROBLEM: Low Bond Precision on  C-C Bonds ...............    0.00808 Ang.
RESPONSE: ...
;
_vrf_PLAT790_lcz043
;
PROBLEM: Centre of Gravity not Within Unit-Cell: Resd.  #          1 Note
RESPONSE: ...
;
_vrf_PLAT910_lcz043
;
PROBLEM: Missing FCF Reflection(s) Below Theta(Min)[Deg]=       3.67 Note
RESPONSE: ...
;
_vrf_PLAT911_lcz043
;
PROBLEM: Missing FCF Refl Between Thmin & STh/L=    0.600        655 Report
RESPONSE: ...
;
_vrf_PLAT972_lcz043
;
PROBLEM: Check Calcd Resid. Dens.  0.80Ang From Ag06           -1.70 eA-3
RESPONSE: ...
;
# end Validation Reply Form
```

It is advisable to attempt to resolve as many as possible of the alerts in all categories. Often the minor alerts point to easily fixed oversights, errors and omissions in your CIF or refinement strategy, so attention to these fine details can be worthwhile. It is up to the individual to critically assess their own results and, if necessary, seek expert advice.

PLATON version of 17/09/2025; check.def file version of 17/09/2025

duplicate check

No duplication found

Datablock lcz043 - ellipsoid plot

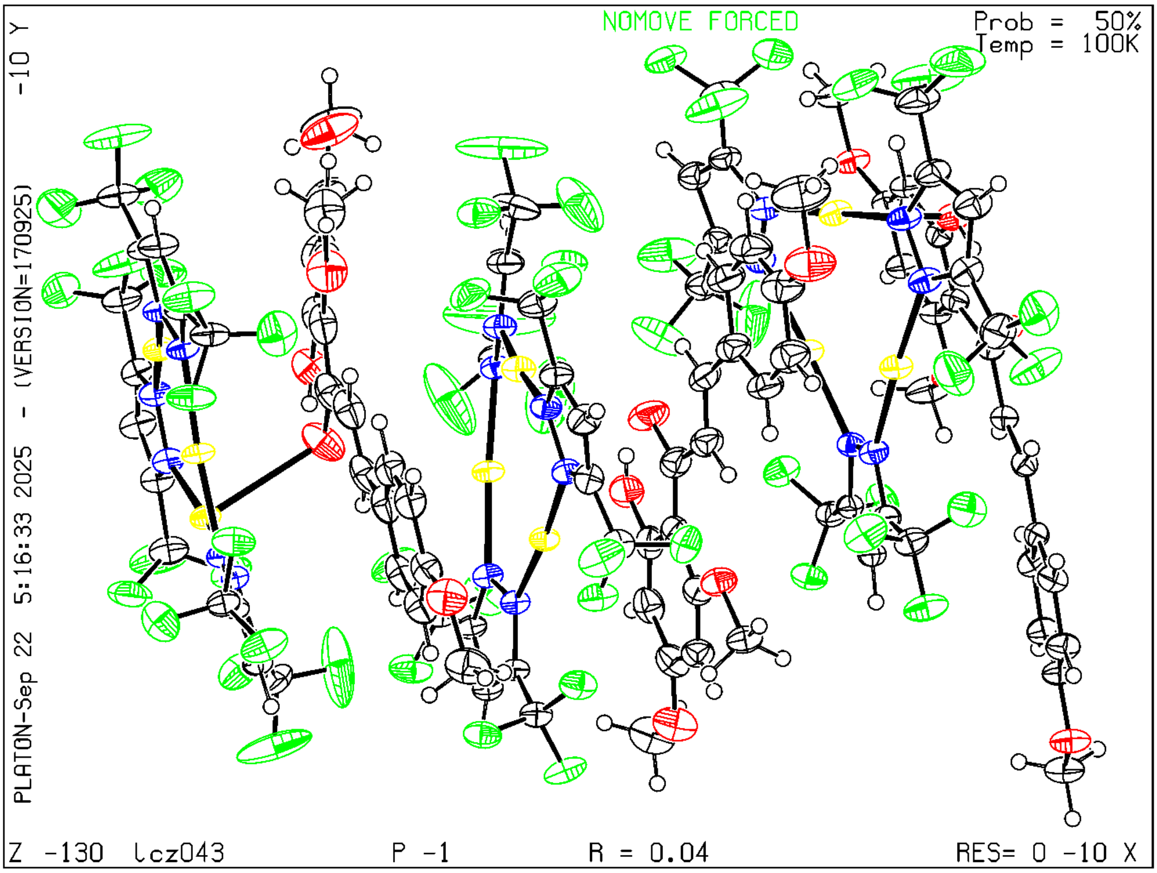

Supplement: Supplementary file 2 — Supporting File 2: anie72462‐sup‐0002‐DataFile.zip. [file ANIE-65-e1218503-s002.zip › supplementary_materials_crystallographic_data submit.zip!/cif_and_checkcif(1-114)/Ag3Pz3_43/Ag3Pz3_43_checkcif.pdf]
